# Herbarium specimens reveal drivers of Arctic shrub growth

**DOI:** 10.1111/nph.70285

**Published:** 2025-06-08

**Authors:** Natalie Iwanycki Ahlstrand, Zoe A. Panchen, Anne D. Bjorkman, James D. M. Speed

**Affiliations:** ^1^ Natural History Museum of Denmark University of Copenhagen Copenhagen 1350 Denmark; ^2^ Department of Biology Acadia University Wolfville B4P 2R6 NS Canada; ^3^ Department of Biological and Environmental Sciences University of Gothenburg Gothenburg 40530 Sweden; ^4^ Department of Natural History, NTNU University Museum Norwegian University of Science and Technology Trondheim 7491 Norway

**Keywords:** Arctic climate change, digital herbarium specimens, *Salix*, shrub growth, shrubification, willows

## Disclaimer

The New Phytologist Foundation remains neutral with regard to jurisdictional claims in maps and in any institutional affiliations.

## Introduction

The Arctic biome is experiencing climate change at rates higher than anywhere else in the world (Jansen *et al*., 2020). Longer, warmer growing seasons have driven increases in plant height, biomass, and changes in distribution and abundance, including an expansion of shrub species (‘shrubification’), ultimately affecting vegetation community dynamics and carbon sequestration across the tundra (Myers‐Smith *et al*., 2011, 2020; Bjorkman *et al*., 2018; Collins *et al*., 2021; Kemppinen *et al*., 2021). However, there is heterogeneity among sites and among species with some sites and some species show little or no response to warming (Elmendorf *et al*., 2012; Myers‐Smith *et al*., 2015a; Bjorkman *et al*., 2020). Thus, understanding when and why some sites or species show dramatic responses to warming, while others less so, is critical to predicting the effects of future climate change on vegetation and associated ecosystem functions. However, existing time‐series research, such as the long‐term International Tundra Experiment (ITEX) established in 1990 (Molau & Mølgaard, 1996; Henry *et al*., 2022), is based on relatively short periods compared to the onset of anthropogenic global warming (AGW) 150–200 years ago (Franzke, 2014).

Arctic shrubs provide a record of growth spanning many decades of environmental change. Radial growth (annual ring widths) has most commonly been used as an indicator of shrub performance over time, and to investigate climate impacts on vegetation processes, including climate reconstructions (Weijers *et al*., 2010; Buchwal *et al*., 2013), climate sensitivity (Myers‐Smith *et al*., 2015a), and interactions with other drivers including herbivory (Vuorinen *et al*., 2022). Axial growth (annual stem growth increments), measured as the distance between winter bud scars or wintermarksepta, also reflects annual variation in growth (Myers‐Smith *et al*., 2015a). While comparatively few studies have included stem increments, just like growth rings, stem increment measurements can also be used as climate proxies (Weijers *et al*., 2010) and to investigate changes in biomass and temperature sensitivities for Arctic shrub species (Hallinger *et al*., 2010; Le Moullec *et al*., 2019).

Herbarium collections of Arctic plants have tremendous potential to be used as an extension of shrub growth chronologies. Herbarium specimens have been used to study the long‐term impacts of climate change on plant function and structure (Panchen & Gorelick, 2017; Speed *et al*., 2022; Ahlstrand *et al*., 2023, 2026). While the interest in the application of herbarium specimens for studying global change has increased in recent years (Lang *et al*., 2019), the exploration of plant functional traits from herbarium specimens is still very much in its infancy (Heberling, 2022). Woody stems can be cut from mounted herbarium sheets and prepared to reveal tree ring chronologies, and historical specimens can greatly expand the timelines of shrub chronologies (Opała‐Owczarek & Owczarek, 2023). However, this includes undesirable destructive sampling of historical records and rare specimens. Here, we demonstrate that digital images of herbarium specimens can be leveraged as a new resource to nondestructively measure plant growth in Arctic shrubs. World‐wide digitisation efforts are increasingly making herbarium specimens and their associated biological and ecological data widely available, including hundreds of thousands of specimens collected from across the Arctic biome (GBIF, 2025).

**Fig. 1:**
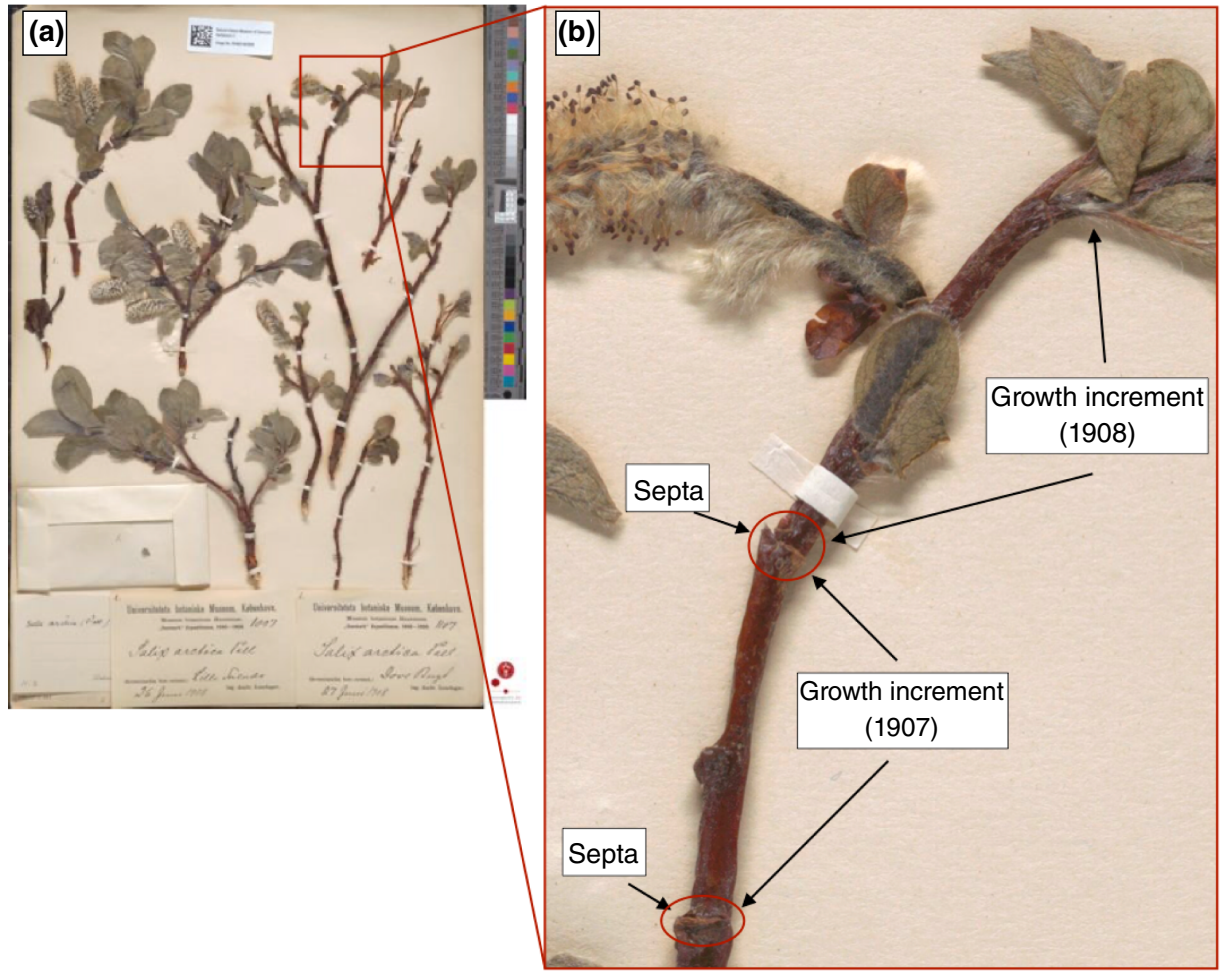
Herbarium specimens of woody plants capture taxonomic, biogeographic, and ecological data, including chronologies of annual growth that can be used to study shrub growth in response to enviromental drivers. (a) Arctic willow (*Salix arctica*) herbarium specimen image collected in Greenland in 1908, with inset (b) showing septa (terminal bud scars) used to measure annual growth increments. On this specimen image, multiple years of growth are detectable providing growth increment data dating back several years.

We investigate a novel derivation of trait data obtained directly from digital images of herbarium specimens to further our understanding of Arctic shrubification and global change: incremental annual stem length growth. Herbarium specimens of many woody plants uniquely document annual growth increments. Arctic shrub growth is relatively slow with very short annual increments; thus, multiple years of growth preceding the collection date can putatively be measured along the stem from a single specimen. For example, a specimen of Arctic willow (*Salix arctica* Pall.) collected in northern Greenland in 1908 could allow us to nondestructively measure annual growth increments dating back to 1899 (Fig. 1). Thus, a single herbarium specimen can provide a chronology of historical annual growth rather than a snapshot in time such as flowering time or leaf traits, providing us with repeated measurements in nature from the same individual and providing a better understanding of interannual effects of climate on Arctic plant growth and development.

We use 482 Arctic specimens of four species in the genus *Salix* L. (the willows) collected in Greenland over 160 years to: (1) develop an approach for quantifying stem incremental growth chronologies from digitised herbarium specimens of Arctic shrubs and assess the feasibility and variability at the stem and individual level; (2) determine how Arctic shrub stem growth has changed over time; and (3) determine whether shrub growth is related to the summer temperature of the growing year based on shrub growth quantified from Greenlandic shrub willow specimens. We discuss collection biases in Arctic specimens that might impact measuring growth chronologies.

## Materials and Methods

### Species and digital specimen selection

The genus *Salix* was selected for this investigation based on the feasibility of detecting and measuring growth increments on stems directly from digital specimens. In addition, the genus is widespread, found in a variety of habitats across the Arctic, and several species have been extensively studied from various location across the Arctic (Gamm *et al*., 2018; Buchwal *et al*., 2020; Boyle *et al*., 2022; Prendin *et al*., 2022). We focused our study using digital specimens from the Natural History Museum of Denmark's Herbarium of the Vascular Plants of Greenland. Four species of differing growth habit, ecology and habitat preferences: *Salix arctica* Pall, *Salix glauca* L., *Salix arctophila* Cockerell, and *Salix herbacea* L. (Supporting Information Table S1) were selected for this investigation, all collected in western Greenland, broadly around the Disko Bay area in phytogeographic region W5 (Fredskild, 1996; Bay *et al*., 2017). We restricted our study to this region based on the large numbers and relatively continuous time series of specimens and the availability of climate data collected in the region dating to 1807 (John, 2021). Digital specimens are available on GBIF (Ahlstrand, 2023, 2025). A total of 482 digital herbarium sheets were assessed for their feasibility in detecting growth increments (Table S1). We excluded specimens where label data were obstructed (and therefore not legible), or for which growth increments were not detectable in digital images due to herbarium conservation or digitisation methods. We excluded specimens that were identified as hybrids based on the specimen labels, specimens that were lacking a year of collection, or specimens for which the most current growth increments were obstructed by leaves or mounting tape, and so on.

### Measuring incremental growth from specimens

Annual growth increments (cm) on stems were measured directly from digital herbarium specimens using ImageJ (Fig. 1) (Rueden *et al*., 2017). Rulers captured in the digital specimen images were used to calibrate the measurement tool in ImageJ. Only stems that exhibited obvious growth in the year of collection (determined by the presence of leaves, flowers, fruit, or opening buds) were selected. Stems with broken, browsed, unopened, or putatively dead buds were not selected as we were unable to confirm the year of growth. We aimed to measure multiple increment chronologies from three different stems on a single herbarium specimen; however, this was not always possible, as in many cases only one or two stems exhibited obvious or unobstructed growth increments. We avoided measuring increments on stems if roots were noted (in case of prostrate species). Each growth increment, beginning from the first full increment corresponding to the year before collection (since growth in the year of collection did not represent a whole growing season), was measured three times from septa to the next septa using the segmented line tool, which allowed for the measurement of curved stem increments, and the average value was used for our analyses. Digital images were annotated using the drawing tool to label stem numbers and increment numbers measured on each specimen. Digital data from specimen labels pertaining to the date of collection and location of sampling were used to refine our final dataset. A growth year was assigned to each annual growth increment.

### Climate variables

Historical climate data for Greenland dating to the 1780s were obtained from the Danish Meteorological Institute for the Weather Station number 4221 in Ilulissat in the Disko Bay region (latitude: 69°14′ N longitude: 51°04′ W; DMI, John, 2021). While these historical climate data lack some information in some years, we chose to use this over other climate data (i.e. CRU TS, Harris *et al*., 2020), as many of the specimens included lacked geographical coordinates. The historical data for this study area show that the average monthly July temperature has been steadily increasing at a rate of 0.1°C per decade (*R* ^2^ = 0.20, *F* = 44.23, *P* < 0.001). Climate data were summarised to create the following climate variables: monthly temperatures and monthly precipitation for the months of January–September in the year of growth and for the months of October, November, and December, and annual mean temperature for the years preceding growth. Annual average temperature in the year of growth and in the year preceding growth were also included. Climatic data were coupled with the corresponding growth year for that increment, and the year preceding growth (growth year − 1) for the months of November, December, and for annual mean temperature. For example, a specimen collected in 1955 with four consecutive growth increments in the years preceding collection was coupled to climate data corresponding to the years 1954, 1953, 1952, and 1950 and with climate data from the preceding year in the case of the winter variables. Temperature anomalies were calculated by subtracting the baseline mean temperature for the period 1961–2010 from the mean annual temperature for a given year, both for the current growth year and for the year preceding growth. Positive anomalies meant that years were classed as warmer than average, whereas negative anomalies meant that the years were cooler than average.

### Temporal and climate growth relationships

All analyses were conducted in R v.4.4.2 (R Core Team, 2023). We used Spearman rank coefficients to evaluate the relationship between the annual growth increments on the first and second stems on the same specimen and to explore the influence of climate on the growth of each *Salix* species. We then fitted generalised additive models (GAMs) separately for each *Salix* species to assess the effects of time and climate variables on annual growth increments using the R package mgcv (Pedersen *et al*., 2019). Annual growth increment was modelled as a function of either time (growth year) or climate predictors (temperature, precipitation, or temperature anomaly in the year preceding growth) using penalised regression splines. To account for variation among specimens (‘individuals’) and stems within specimens (on the same individual), we included random intercepts effects for specimen ID (NHMD_ID) and the nested stem ID (stem_No). This structure allowed us to control for repeated measures within individual specimens while accounting for additional variation at the stem level. Models were fitted using a Gamma distribution with a log‐link function (family = Gamma (link = ‘log’)) as these parameters best fit our data and produced models with the lowest Akaike Information Criterion (AIC) values. Models were evaluated by inspecting standardised residuals, fitted values, and error distributions.

## Results

### Feasibility of approach and variation at the individual and stem level

A total of 482 digital herbarium sheets were assessed for their feasibility in detecting growth increments and included specimens collected between 1833 and 1993. Between 10% and 37% of the specimens we assessed met our criteria for growth increments (*S. arctica* 20%, *S. glauca* 10%, *S. herbacea* 35%, and *S. arctophila* 37%; Table S1).

The number of growth increments ranged from one to nine per stem, and this varied by species, with *S. arctophila* and *S. herbacea* exhibiting greater numbers of increments on average per stem. The mean annual growth increments and variance in growth per species also differed between species, with *S. herbacea* consistently having the smallest incremental growth and lowest variance (mean = 0.36 cm yr^−1^, var = 0.07 cm) and *S. glauca* showing the largest growth (mean = 2.92 cm yr^−1^, var = 3.94 cm) (Table S1). Smaller, prostrate‐growing willows were better candidates for this study. These consistently had fewer leaves obstructing stems, and the shorter annual growth increments meant that a greater number of annual increments could be measured from a single stem, compared to the upright willows and generally more leaf‐dense specimens of *S. glauca*. However, specimen conservation practices such as the use of mounting tape and the positioning of herbarium labels were often what limited specimen use for this work. In addition, we observed many *S. glauca* and *S. arctica* specimens with broken or browsed branches, rendering these specimens unsuitable for measurement.

### Response to time and climate

**Fig. 2:**
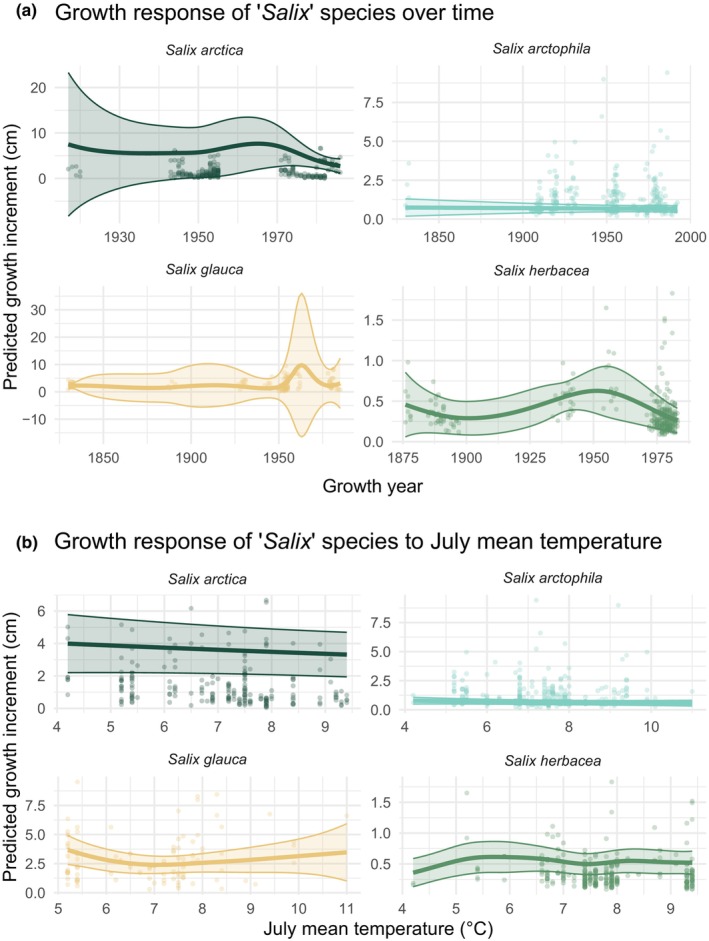
Model estimated trends in growth over time and in response to climate from generalised additive models (GAMs) for each *Salix* species, plotted as the partial effects of (a) growth year and (b) temperature. Shaded ribbons around the fitted lines represent the 95% confidence intervals around the predicted mean growth values from GAMs.

We found a significant nonlinear effect of year on growth increment for *S. herbacea* (estimated degrees of freedom (edf) = 3.65, *F* = 3.72, *P* = 0.01). However, despite substantial variability in growth among years, no significant relationship between year and increment length was found for the other species, indicating that stem growth did not change over time (Fig. 2a; see GAM output in Tables S2–S4). Significant correlations were found between the annual growth increments on the first and second stems measured on the same specimens (Table S5; Fig. S1).

Variance in growth was found to be greater in warmer than average years and in the period post‐1950 for all species (i.e. growth may be more difficult to predict in warmer years and over time due to increased temperature variability in more recent years) (Fig. S2).

Growth increments of *S. glauca* exhibited a significant nonlinear response to mean temperatures in April (edf = 8.05, *F* = 2.55, *P* = 0.02), July (edf = 2.65, *F* = 3.40, *P* = 0.021; Fig. 2b), and August (edf = 1.00, *F* = 6.37, *P* = 0.01), as well as temperature anomaly (edf = 1.00, *F* = 5.16, *P* = 0.026) and mean annual temperatures for the years preceding growth year (edf = 1.00, *F* = 10.35, *P* = 0.002). For *S. herbacea*, mean temperature in April (edf = 1.00, *F* = 4.77, *P* = 0.03) and June (edf = 5.11, *F* = 2.18, *P* = 0.04), temperature anomaly (edf = 1.00, *F* = 7.87, *P* = 0.005), and the mean temperatures in the months of November (edf = 1.86, *F* = 4.41, *P* = 0.01) and December (edf = 1.00, *F* = 4.39, *P* = 0.04) in the year preceding growth year all had significant effects on *S. herbacea* growth increments. Temperature anomaly in the year preceding growth was also found to be a significant factor in the growth of *S. arctophila* (edf = 8.26, *F* = 3.35, *P* = 0.001). No relationships were found between climate variables and growth for *S. arctica* based on our GAMs (Table S2); however, a significant correlation was found between July temperature and growth for this species based on Spearman rank correlation analyses (*P* = 0.03, Table S6).

## Discussion

Our study demonstrates a novel use of digital herbarium specimens: extracting incremental stem measurements to understand interannual variation in the growth of Arctic shrubs. Across four Arctic willow species, we found distinct growth patterns and species‐specific relationships with summer temperature in keeping with other studies that find that local conditions or microhabitat play an important role in explaining growth (Myers‐Smith *et al*., 2015a; Panchen & Gorelick, 2016; Dobbert *et al*., 2022). The approach documented here advances our understanding of the climate sensitivity of shrub growth in the Arctic by quantifying incremental stem growth across many individuals of multiple species and sites, without having to do more costly and time‐consuming field work, and extending chronologies back further than possible with other methods. This demonstrates the potential for further ecological and trait‐based studies based on nondestructive assessments of digital herbarium collections and advances our understanding of Arctic vegetation responses to climate through the Anthropocene.

Although all our samples were from the phytogeographic region around the Disko Bay area, the between‐individual variation in growth was high. This may be due to high local‐scale habitat heterogeneity in Arctic tundra ecosystems (Elmendorf *et al*., 2012) or to genotypic differences among individuals. Further substantial variation was found between branches of the same specimens (and this level varies from species to species), suggesting other factors contribute to growth such as branch position, exposure, or stress. The high variation between branches and individuals (as also summarised by Myers‐Smith *et al*., 2015b) necessitates the measurement of multiple branches per specimen and multiple individuals per locality and date, where feasible. The ongoing digitisation of herbarium specimens around the world facilitates greater sample depth, and combining specimens across collections would reduce spatial bias (but potentially introduce spatial variation).

We found the growth of only one species, *S. herbacea*, to significantly change over time; otherwise, no changes were found in growth over time when accounting for the random effects, which is consistent with other studies (Myers‐Smith *et al*., 2015a; Bjorkman *et al*., 2020) and may indicate that the growth of some shrub species is stable or robust under climate change (Franzke, 2014; Panchen *et al*., 2022).

Species‐specific ecological strategies, such as habitat preference and growth habit, likely play a role in the species‐specific climate sensitivities observed here (Table S1). Microhabitat, such as wetter areas (Jørgensen *et al*., 2015; Ackerman *et al*., 2017; Power *et al*., 2024) or areas of deeper snow cover or nutrient‐rich areas (Prendin *et al*., 2022), can possibly drive the within‐species variations in annual growth we observed.

*Salix glauca* was the only species we found to have a positive relationship with mean July temperature. *Salix glauca*, is a larger, upright willow that prefers drier, nutrient‐poor sites, and it is associated with greening in the Arctic (Young *et al*., 2016). The nonlinear relationship we found between growth and mean temperature in July for this willow is in keeping with Elmendorf *et al*. (2012) and Myers‐Smith *et al*. (2015a) where taller, upright willows were found to be more sensitive to climate. Past studies in Western and Southern Greenland underscore that warm summer temperature is a critical driver for *Salix* radial growth, with *S. glauca* in particular exhibiting greater radial growth in warmer years (Jørgensen *et al*., 2015; Prendin *et al*., 2022). However, while increasing July temperatures generally enhance growth, our results show that growth appears to increase with temperature up to *c*. 7–8°C before levelling off or declining slightly, indicating a potential thermal optimum. This also matches patterns found in radial growth for the species in Western Greenland (Jørgensen *et al*., 2015; Gamm *et al*., 2018). As also found in ring‐width studies, the herbarium data show a clear positive relationship between summer temperatures and annual stem growth, confirming that warmer growing seasons consistently enhance both radial and axial growth of *S. glauca* in West Greenland and that radial and axial growth taper off at temperatures exceeding 7°C.

*Salix herbacea* is considered a small, trailing snow bed shrub. Its growth limitations may be influenced more by the timing of spring snowmelt and temperatures early in the season, as shown in our study with the species responding to temperatures in April and June. Radial growth rings for *S. herbacea* in Iceland revealed that both temperature and precipitation drive the species' growth and that drivers of growth vary by sampling site (Phulara *et al*., 2022). In the European Alps, Wheeler *et al*. (2016) found that earlier snowmelt and longer growing seasons reduced the performance of *S. herbacea*. *Salix arctica* growth increments showed no response to climatic variables in our study, which may be indicative that the species is more stress tolerant in Western Greenland. *Salix arctica* is one of the most widely distributed Arctic willows, and because it can grow in a range of hydrological conditions, it may be more resilient to change (Dawson & Bliss, 1989). However, in high Arctic Greenland, radial growth of *S. arctica* was found to be sensitive to summer temperatures (Buchwal *et al*., 2019). *Salix arctophila* is a species found in wet tundra and stream margins, and while studies on this taxon are limited, its growth could conceivably be more dependent on hydrological changes than temperature (Myers‐Smith *et al*., 2015a; Ackerman *et al*., 2017; Phulara *et al*., 2022).

### Limitations and biases

Although there are some gaps in the time series dataset of *Salix* shrub growth assembled here (Fig. S1), the chronology within a single phytogeographic region offers temporal coverage better than many other Arctic regions due to recognised collection biases (Daru *et al*., 2018; Panchen *et al*., 2019). We note that our herbarium data lack a critical time period in terms of global change (our most recent specimens were collected in the early 1990s). However, the growth specimen data we obtained in this study capture nearly the full range of interannual climate variability and highs and lows recorded between the years 1833 and 1993 (John, 2021), corresponding to our samples from Western Greenland, and this is critical for modelling the effects of climate on growth. While these critical modern years of climate warming were not possible to obtain from these specimens, the herbarium data do compliment modern field sampling, which started in the 1990s through the ITEX initiative.

### Future directions

Our study serves as a proof of concept and methodological template for investigating historical stem incremental growth from shrubs across the Arctic biome. There are currently close to 30 000 digital specimen images from across the Arctic biome on GBIF (GBIF, 2025). Future directions of promise include combining stem elongation growth and stem radial growth chronologies in integrated analyses as well as linking growth to interannual phenological dynamics. There is also potential for associating individual growth rates with specimen genotyping through museomics. The continued collection of shrub specimens in natural history collections is needed to continue to contribute to understanding the long‐term processes of vegetation response to climate change. Specimen mounting techniques can be adapted to facilitate trait‐based studies: for example, sewing specimens with string onto herbarium sheets is better than tape, and thin tape strips are better than thick. While AI and machine learning have been largely not included in shrub studies, we are seeing rapid advances including automatic ring detection in shrub cross‐sections (Gillert *et al*., 2023) and such advances in machine learning could make extracting data from the digitised herbarium specimens even more efficient in the future (Jones *et al*., 2024). Computer vision has been used for quantifying traits across herbarium specimens including flowering and leaf area (Weaver & Smith, 2023). If models could be trained to extract stem growth increments, the potential for analysis of growth trends from herbaria would be increased.

## Conclusions

Digital herbaria are untapped resources for studying chronologies of plant growth. Their application can contribute to the generation of new knowledge about plant growth dynamics at greater temporal, spatial, and taxonomic scales than any other source of data. Herbarium specimens complement and build upon locally based studies by extending timelines and populations included to improve our understanding of how shrubs respond to climate change over the whole period of AGW.

## Competing interests

None declared.

## Author contributions

NIA conceived the study, designed the methods, collected and analysed data, and led the writing of the manuscript. ZP, AB, and JDMS, all contributed to the interpretation of the results and to the writing and reviewing of the manuscript.

## Supporting information


**Fig. S1** Relationship between growth increments on first and second stems (stem 1 and stem 2) from stems on the same specimen for *Salix* species.
**Fig. S2** Variability in growth increments over time for *Salix* species.
**Table S1** *Salix* species collected in the Disko Bay phytogeographic region of Western Greenland.
**Table S2** Results of generalised additive models (GAMs).
**Table S3** Variance explained for each fitted GAM.
**Table S4** Model metrics from GAMs.
**Table S5** Spearman rank correlation results for annual growth increments on the first and second stems from the same specimen for *Salix* species.
**Table S6** Spearman rank correlation analyses of annual growth increment and mean temperature in July corresponding to the year of growth.
Please note: Wiley is not responsible for the content or functionality of any Supporting Information supplied by the authors. Any queries (other than missing material) should be directed to the *New Phytologist* Central Office.

## Data Availability

*Salix* specimens used in this study are publicly available on the Global Biodiversity Information Facility (GBIF) at doi: 10.15468/dl.5syf6k. The accession numbers of the specimens used in the study and growth measurements can be found on Zenodo at doi: 10.5281/zenodo.15489625.
