# Supplementary material for: Herbarium specimens reveal drivers of Arctic shrub growth
Source: New Phytol. 2025 Jun 8;251(2):602–9. doi: 10.1111/nph.70285 (PMC13278643; doi:10.1111/nph.70285)
Supplement: Supplementary file 1 — Fig. S1 Relationship between growth increments on first and second stems (stem 1 and stem 2) from stems on the same specimen for Salix species. Fig. S2 Variability in growth increments over time for Salix species. Table S1 Salix species collected in the Disko Bay phytogeographic region of Western Greenland. Table S2 Results of generalised additive models (GAMs). Table S3 Variance explained for each fitted GAM. Table S4 Model metrics from GAMs. Table S5 Spearman rank correlation results for annual growth increments on the first and second stems from the same specimen for Salix species. Table S6 Spearman rank correlation analyses of annual growth increment and mean temperature in July corresponding to the year of growth. Please note: Wiley is not responsible for the content or functionality of any Supporting Information supplied by the authors. Any queries (other than missing material) should be directed to the New Phytologist Central Office. [file NPH-251-602-s001.pdf]

New Phytologist Supporting Information

Article title: Herbarium specimens reveal drivers of Arctic shrub growth

Authors: Natalie Iwanycki Ahlstrand, Zoe A. Panchen, Anne D. Bjorkman, James DM Speed

Article acceptance date: 17 May 2025

Contents

Supporting Tables:

Table S1: *Salix* species collected in the Disko Bay phytogeographic region of Western Greenland.

Table S2: Results of Generalized Additive Models (GAMs)

Table S3. Variance explained for each fitted GAM

Table S4. Model metrics from GAMs

Table S5. Spearman Rank Correlation results for annual growth increments on the first and second stems from the same specimens for *Salix* species

Table S6. Spearman Rank Correlation Analyses of annual growth increment and mean temperature in July corresponding to the year of growth

Supporting Figures:

Figure S1. Relationship between annual growth increments on the first and second stems from the same specimens for *Salix* species

Figure S2. Variability in growth increments over time for *Salix* species

Table S1. *Salix* species used in this study. Specimens were collected in the Disko Bay phytogeographic region of Western Greenland and are available on the Global Biodiversity Information Facility (GBIF).

Species	Habit / Growth form[1]	Habitat	N. of specimens from greater Disko Bay area	N. of specimens meeting criteria for growth increments	Specimen date range	Total number of growth increments measured	Mean growth increment length ± SD	Variance in growth pre1950 / pos1950
Salix arctica Pall	Prostrate, mat-forming, sometimes upright	Wet to dry Arctic-alpine environments	122	22	1921–1987	189	1.50 ± 1.36	2.08 / 1.80
Salix arctophila Cockerell	Prostrate, Low to medium shrub (10–50 cm)	Hummocks within wet mossy meadows, grass or sedge meadows, and along stream or pond margins	76	28	1833–1993	227	1.45 ± 1.29	2.06 / 1.40
Salix glauca L.	Upright shrub (0.5–2m tall)	Heaths and inland tundra areas	183	18	1834–1986	103	2.92 ± 1.99	1.35 / 5.45
Salix herbacea L.	Prostrate, Tiny, creeping mats (1–6 cm)	Snowbeds, moist tundra, heaths	101	22	1879 - 1984	266	0.36 ± 0.26	0.04 / 0.08

[1]Species ecology and habitat information from Flora of North America; Böcher, T.W., Fredskild, B., Holmen, K., and Jakobsen, K. 1978. Grønlands Flora (3. ed.). Copenhagen. 312 pp.

[2] Digitigal specimens from the Greenland Vascular Plant Herbarium at the Natural History Museum of Denmark (NHMD) are available on GBIF (https://www.gbif.org/dataset/0d1f9cee-7cb7-4d3a-a8c4-d2ca6edcd23b) and through the NHMD's collection portal (https://collections.snm.ku.dk/en).

Table S2. Results of Generalized Additive Models (GAMs) modeling growth increment as a function of either time (growth year) or climate predictors (temperature, precipitation, or temperature anomaly in the year preceding growth). Models included random intercepts effects for specimen ID (NHMD_ID) and the nested stem ID (stem_No), and models were fitting using a Gamma distribution with a log-link function (family = Gamma (link = “log”). Significance levels: * p < 0.05, ** p < 0.01, *** p < 0.001.

Model	Predictor	edf	F	p-value	Significance level
S_arctica_julAveTemp	julAveTemp	1.00	1.62	0.204863561	
S_arctica_julAveTemp	NHMD_ID	16.47	46.17	0	
S_arctica_julAveTemp	NHMD_ID/STEM	15.97	2.36	0.038547531	
S_arctica_junAveTemp	junAveTemp	1.00	1.64	0.202987888	
S_arctica_junAveTemp	NHMD_ID	16.44	55.05	0	
S_arctica_junAveTemp	NHMD_ID/STEM	15.90	2.31	0.044175626	
S_arctica_mayAveTemp	mayAveTemp	1.00	0.05	0.832201703	
S_arctica_mayAveTemp	NHMD_ID	16.36	57.24	0	
S_arctica_mayAveTemp	NHMD_ID/STEM	16.18	2.66	0.025749478	
S_arctica_aprAveTemp	aprAveTemp	1.00	0.12	0.72682934	
S_arctica_aprAveTemp	NHMD_ID	16.36	57.56	0	
S_arctica_aprAveTemp	NHMD_ID/STEM	16.20	2.80	0.013441502	
S_arctica_marAveTemp	marAveTemp	1.00	0.00	0.992089654	
S_arctica_marAveTemp	NHMD_ID	16.33	57.18	0	
S_arctica_marAveTemp	NHMD_ID/STEM	16.17	2.76	0.017675311	
S_arctica_febAveTemp	febAveTemp	3.38	1.03	0.37538313	
S_arctica_febAveTemp	NHMD_ID	15.93	53.93	0	
S_arctica_febAveTemp	NHMD_ID/STEM	16.58	2.63	0.014715289	
S_arctica_janAveTemp	janAveTemp	1.00	0.07	0.785219614	
S_arctica_janAveTemp	NHMD_ID	16.37	55.51	0	
S_arctica_janAveTemp	NHMD_ID/STEM	16.13	2.68	0.017838168	
S_arctica_augAveTemp	augAveTemp	1.00	0.42	0.517040638	
S_arctica_augAveTemp	NHMD_ID	16.40	55.19	0	
S_arctica_augAveTemp	NHMD_ID/STEM	16.15	2.69	0.019492457	
S_arctica_octAveTemp.1	octAveTemp.1	1.23	2.19	0.184799974	
S_arctica_octAveTemp.1	NHMD_ID	16.44	51.25	0	
S_arctica_octAveTemp.1	NHMD_ID/STEM	15.97	2.32	0.033038238	
S_arctica_novAveTemp.1	novAveTemp.1	1.00	0.05	0.824542178	
S_arctica_novAveTemp.1	NHMD_ID	16.34	58.10	0	
S_arctica_novAveTemp.1	NHMD_ID/STEM	16.23	2.70	0.0163807	
S_arctica_decAveTemp.1	decAveTemp.1	1.00	4.19	0.042413339	
S_arctica_decAveTemp.1	NHMD_ID	16.35	54.73	0	
S_arctica_decAveTemp.1	NHMD_ID/STEM	16.23	2.69	0.022405286	
S_arctica_annualAveTemp	annualAveTemp	1.00	0.01	0.914980637	
S_arctica_annualAveTemp	NHMD_ID	16.43	52.37	0	
S_arctica_annualAveTemp	NHMD_ID/STEM	16.14	2.56	0.029980684	

S_arctica_Anomaly	Anomaly	1.26	0.17	0.86083915	
S_arctica_Anomaly	NHMD_ID	16.21	57.22	0	
S_arctica_Anomaly	NHMD_ID/STEM	16.35	2.77	0.018820663	
S_arctica_annualAveTemp.1	annualAveTemp.1	1.00	0.08	0.782264813	
S_arctica_annualAveTemp.1	NHMD_ID	16.33	49.16	0	
S_arctica_annualAveTemp.1	NHMD_ID/STEM	16.16	2.44	0.037934119	
S_arctica_Anomaly.1	Anomaly.1	1.00	0.28	0.599562469	
S_arctica_Anomaly.1	NHMD_ID	16.21	58.15	0	
S_arctica_Anomaly.1	NHMD_ID/STEM	16.26	2.87	0.015429744	
S_arctica_janPrecip	janPrecip	1.35	1.49	0.370660078	
S_arctica_janPrecip	NHMD_ID	16.34	44.26	0	
S_arctica_janPrecip	NHMD_ID/STEM	15.04	1.86	0.049603554	
S_arctica_febPrecip	febPrecip	1.00	0.30	0.583883125	
S_arctica_febPrecip	NHMD_ID	16.88	54.15	0	
S_arctica_febPrecip	NHMD_ID/STEM	15.88	2.56	0.091615769	
S_arctica_marPrecip	marPrecip	1.00	0.65	0.422361907	
S_arctica_marPrecip	NHMD_ID	16.97	47.30	0	
S_arctica_marPrecip	NHMD_ID/STEM	15.78	2.29	0.139163026	
S_arctica_aprPrecip	aprPrecip	1.35	1.63	0.12625013	
S_arctica_aprPrecip	NHMD_ID	16.35	45.23	0	
S_arctica_aprPrecip	NHMD_ID/STEM	15.11	2.06	0.023650969	
S_arctica_mayPrecip	mayPrecip	1.00	0.44	0.507623876	
S_arctica_mayPrecip	NHMD_ID	16.59	48.84	0	
S_arctica_mayPrecip	NHMD_ID/STEM	14.71	2.01	0.02701304	
S_arctica_junPrecip	junPrecip	1.00	0.00	0.988019086	
S_arctica_junPrecip	NHMD_ID	16.57	47.31	0	
S_arctica_junPrecip	NHMD_ID/STEM	14.74	2.06	0.027361899	
S_arctica_julPrecip	julPrecip	1.00	3.08	0.081334136	
S_arctica_julPrecip	NHMD_ID	16.79	43.74	0	
S_arctica_julPrecip	NHMD_ID/STEM	14.00	1.85	0.016810464	
S_arctica_augPrecip	augPrecip	1.00	0.22	0.638043665	
S_arctica_augPrecip	NHMD_ID	16.56	48.40	0	
S_arctica_augPrecip	NHMD_ID/STEM	14.66	2.14	0.018840842	
S_arctica_annualPrecip	annualPrecip	1.00	0.04	0.837902736	
S_arctica_annualPrecip	NHMD_ID	17.13	56.28	0	
S_arctica_annualPrecip	NHMD_ID/STEM	16.20	3.26	0.044000986	
S_arctica_growth_year	growth_year	3.67	1.73	0.127892646	
S_arctica_growth_year	NHMD_ID	17.27	52.58	0	
S_arctica_growth_year	NHMD_ID/STEM	15.48	1.52	0.450332909	
S_arctophila_julAveTemp	julAveTemp	1.33	1.06	0.242127742	
S_arctophila_julAveTemp	NHMD_ID	14.91	11.87	1.19598E-05	
S_arctophila_julAveTemp	NHMD_ID/STEM	33.87	2.38	0.00088344	
S_arctophila_junAveTemp	junAveTemp	1.00	0.00	0.982753997	
S_arctophila_junAveTemp	NHMD_ID	14.70	11.45	2.31601E-05	
S_arctophila_junAveTemp	NHMD_ID/STEM	34.20	2.52	0.000515316	

S_arctophila_mayAveTemp	mayAveTemp	2.42	0.58	0.610882945	
S_arctophila_mayAveTemp	NHMD_ID	15.75	11.01	3.00928E-05	
S_arctophila_mayAveTemp	NHMD_ID/STEM	34.89	2.51	0.000551333	
S_arctophila_aprAveTemp	aprAveTemp	1.74	4.51	0.010705402	
S_arctophila_aprAveTemp	NHMD_ID	15.54	12.00	2.32891E-05	
S_arctophila_aprAveTemp	NHMD_ID/STEM	36.31	2.82	0.000383122	
S_arctophila_marAveTemp	marAveTemp	2.53	1.14	0.31265159	
S_arctophila_marAveTemp	NHMD_ID	15.14	9.76	8.57631E-05	
S_arctophila_marAveTemp	NHMD_ID/STEM	35.78	2.44	0.000619709	
S_arctophila_febAveTemp	febAveTemp	2.08	2.60	0.091903097	
S_arctophila_febAveTemp	NHMD_ID	15.10	9.16	9.30258E-05	
S_arctophila_febAveTemp	NHMD_ID/STEM	35.01	2.25	0.000937504	
S_arctophila_janAveTemp	janAveTemp	1.00	0.72	0.397174098	
S_arctophila_janAveTemp	NHMD_ID	16.36	11.05	3.0809E-05	
S_arctophila_janAveTemp	NHMD_ID/STEM	34.11	2.33	0.001781201	
S_arctophila_augAveTemp	augAveTemp	2.26	1.88	0.124914564	
S_arctophila_augAveTemp	NHMD_ID	14.54	10.92	4.09661E-05	
S_arctophila_augAveTemp	NHMD_ID/STEM	34.62	2.36	0.000905256	
S_arctophila_octAveTemp.1	octAveTemp.1	1.00	0.78	0.379720105	
S_arctophila_octAveTemp.1	NHMD_ID	15.04	10.42	2.45967E-05	
S_arctophila_octAveTemp.1	NHMD_ID/STEM	35.33	2.54	0.000320535	
S_arctophila_novAveTemp.1	novAveTemp.1	1.00	1.26	0.263751127	
S_arctophila_novAveTemp.1	NHMD_ID	14.99	9.95	2.33034E-05	
S_arctophila_novAveTemp.1	NHMD_ID/STEM	34.85	2.41	0.000365499	
S_arctophila_decAveTemp.1	decAveTemp.1	2.97	0.90	0.368120998	
S_arctophila_decAveTemp.1	NHMD_ID	15.06	9.75	4.49485E-05	
S_arctophila_decAveTemp.1	NHMD_ID/STEM	35.46	2.30	0.000694399	
S_arctophila_annualAveTemp	annualAveTemp	1.00	0.12	0.734145247	
S_arctophila_annualAveTemp	NHMD_ID	15.83	12.25	5.8224E-05	
S_arctophila_annualAveTemp	NHMD_ID/STEM	33.76	2.51	0.002035833	
S_arctophila_Anomaly	Anomaly	2.44	2.14	0.097998101	
S_arctophila_Anomaly	NHMD_ID	14.22	10.59	6.1213E-05	
S_arctophila_Anomaly	NHMD_ID/STEM	34.98	2.49	0.000563772	
S_arctophila_annualAveTemp.1	annualAveTemp.1	1.67	2.48	0.089773692	
S_arctophila_annualAveTemp.1	NHMD_ID	12.98	8.44	0.000199218	
S_arctophila_annualAveTemp.1	NHMD_ID/STEM	35.81	2.14	0.001376584	
**S_arctophila_Anomaly.1**	**Anomaly.1**	**8.28**	**3.35**	**0.001022479**	**
S_arctophila_Anomaly.1	NHMD_ID	9.23	4.78	0.003226902	
S_arctophila_Anomaly.1	NHMD_ID/STEM	40.09	3.05	8.62721E-07	
S_arctophila_janPrecip	janPrecip	1.00	2.52	0.114380466	
S_arctophila_janPrecip	NHMD_ID	10.68	8.97	0.000416245	
S_arctophila_janPrecip	NHMD_ID/STEM	36.65	3.17	5.44792E-05	
S_arctophila_febPrecip	febPrecip	1.23	0.21	0.609474875	
S_arctophila_febPrecip	NHMD_ID	10.02	8.55	0.000268615	
S_arctophila_febPrecip	NHMD_ID/STEM	35.47	3.15	5.38926E-05	

S_arctophila_marPrecip	marPrecip	1.00	0.75	0.388551872	
S_arctophila_marPrecip	NHMD_ID	9.70	8.09	0.000323874	
S_arctophila_marPrecip	NHMD_ID/STEM	35.64	3.22	2.90837E-05	
S_arctophila_aprPrecip	aprPrecip	2.07	1.77	0.207751859	
S_arctophila_aprPrecip	NHMD_ID	9.12	7.56	0.000720671	
S_arctophila_aprPrecip	NHMD_ID/STEM	36.71	3.05	3.57123E-05	
S_arctophila_mayPrecip	mayPrecip	6.04	1.35	0.224440033	
S_arctophila_mayPrecip	NHMD_ID	7.67	5.63	0.002090564	
S_arctophila_mayPrecip	NHMD_ID/STEM	38.08	3.45	1.75966E-06	
S_arctophila_junPrecip	junPrecip	1.00	0.33	0.567674172	
S_arctophila_junPrecip	NHMD_ID	9.83	8.20	0.00069529	
S_arctophila_junPrecip	NHMD_ID/STEM	35.54	3.16	3.24592E-05	
S_arctophila_julPrecip	julPrecip	1.00	2.83	0.094607004	
S_arctophila_julPrecip	NHMD_ID	9.55	8.58	0.00080383	
S_arctophila_julPrecip	NHMD_ID/STEM	36.75	3.28	7.21015E-05	
S_arctophila_augPrecip	augPrecip	1.00	4.40	0.037661101	
S_arctophila_augPrecip	NHMD_ID	9.00	7.44	0.001738693	
S_arctophila_augPrecip	NHMD_ID/STEM	37.32	3.30	4.31126E-05	
S_arctophila_annualPrecip	annualPrecip	1.00	0.08	0.775993199	
S_arctophila_annualPrecip	NHMD_ID	10.58	10.94	0.000295052	
S_arctophila_annualPrecip	NHMD_ID/STEM	33.93	3.46	0.000177172	
S_arctophila_growth_year	growth_year	1.00	0.10	0.755062224	
S_arctophila_growth_year	NHMD_ID	14.88	10.11	2.91629E-05	
S_arctophila_growth_year	NHMD_ID/STEM	34.58	2.45	0.000386497	
S_herbacea_julAveTemp	julAveTemp	4.61	1.00	0.429667097	
S_herbacea_julAveTemp	NHMD_ID	15.36	20.55	0.000351264	
S_herbacea_julAveTemp	NHMD_ID/STEM	27.17	2.28	0.287875364	
**S_herbacea_junAveTemp**	**junAveTemp**	**5.11**	**2.18**	**0.04356253**	*
S_herbacea_junAveTemp	NHMD_ID	15.29	24.53	6.66112E-05	
S_herbacea_junAveTemp	NHMD_ID/STEM	27.56	2.84	0.128282442	
S_herbacea_mayAveTemp	mayAveTemp	2.44	0.55	0.616148722	
S_herbacea_mayAveTemp	NHMD_ID	15.84	25.55	9.56617E-05	
S_herbacea_mayAveTemp	NHMD_ID/STEM	27.11	2.62	0.275666397	
**S_herbacea_aprAveTemp**	**aprAveTemp**	**1.00**	**4.77**	**0.030016707**	*
S_herbacea_aprAveTemp	NHMD_ID	15.96	29.42	2.58503E-05	
S_herbacea_aprAveTemp	NHMD_ID/STEM	27.16	3.05	0.213377338	
S_herbacea_marAveTemp	marAveTemp	1.00	2.30	0.13050496	
S_herbacea_marAveTemp	NHMD_ID	15.74	27.82	2.94301E-05	
S_herbacea_marAveTemp	NHMD_ID/STEM	27.18	3.01	0.184813106	
S_herbacea_febAveTemp	febAveTemp	1.00	3.86	0.050556781	
S_herbacea_febAveTemp	NHMD_ID	15.94	28.90	2.09572E-05	
S_herbacea_febAveTemp	NHMD_ID/STEM	27.01	2.99	0.200269901	
S_herbacea_janAveTemp	janAveTemp	1.89	7.29	0.000572252	
S_herbacea_janAveTemp	NHMD_ID	15.15	27.68	3.99715E-05	
S_herbacea_janAveTemp	NHMD_ID/STEM	28.14	3.42	0.091826014	

S_herbacea_augAveTemp	augAveTemp	7.13	1.83	0.068141016	
S_herbacea_augAveTemp	NHMD_ID	14.52	21.98	0.000687017	
S_herbacea_augAveTemp	NHMD_ID/STEM	28.94	2.96	0.174995059	
S_herbacea_octAveTemp.1	octAveTemp.1	4.91	1.67	0.142125965	
S_herbacea_octAveTemp.1	NHMD_ID	15.87	24.25	0.00051715	
S_herbacea_octAveTemp.1	NHMD_ID/STEM	27.65	2.48	0.39519001	
**S_herbacea_novAveTemp.1**	**novAveTemp.1**	**1.86**	**4.41**	**0.011194011**	*
S_herbacea_novAveTemp.1	NHMD_ID	15.85	27.35	5.71536E-05	
S_herbacea_novAveTemp.1	NHMD_ID/STEM	27.12	2.92	0.228012741	
**S_herbacea_decAveTemp.1**	**decAveTemp.1**	**1.00**	**4.39**	**0.037081595**	*
S_herbacea_decAveTemp.1	NHMD_ID	15.76	28.24	4.11591E-05	
S_herbacea_decAveTemp.1	NHMD_ID/STEM	27.28	3.07	0.204099887	
S_herbacea_annualAveTemp	annualAveTemp	4.00	1.99	0.077291561	
S_herbacea_annualAveTemp	NHMD_ID	16.02	29.56	4.66494E-05	
S_herbacea_annualAveTemp	NHMD_ID/STEM	27.50	2.95	0.215629388	
**S_herbacea_Anomaly**	**Anomaly**	**1.00**	**7.87**	**0.005477722**	**
S_herbacea_Anomaly	NHMD_ID	18.14	37.26	8.62676E-05	
S_herbacea_Anomaly	NHMD_ID/STEM	26.10	2.59	0.748909084	
S_herbacea_annualAveTemp.1	annualAveTemp.1	6.12	2.03	0.050846987	
S_herbacea_annualAveTemp.1	NHMD_ID	15.77	29.35	5.46217E-05	
S_herbacea_annualAveTemp.1	NHMD_ID/STEM	27.84	2.97	0.213382644	
S_herbacea_Anomaly.1	Anomaly.1	2.66	0.69	0.47779175	
S_herbacea_Anomaly.1	NHMD_ID	15.15	23.92	0.000127654	
S_herbacea_Anomaly.1	NHMD_ID/STEM	27.41	2.75	0.204613604	
S_herbacea_janPrecip	janPrecip	2.64	2.66	0.048970902	
S_herbacea_janPrecip	NHMD_ID	15.17	25.65	1.85158E-05	
S_herbacea_janPrecip	NHMD_ID/STEM	24.13	2.31	0.256745876	
S_herbacea_febPrecip	febPrecip	1.76	0.74	0.468120271	
S_herbacea_febPrecip	NHMD_ID	14.89	26.60	4.68397E-05	
S_herbacea_febPrecip	NHMD_ID/STEM	24.10	2.59	0.231831327	
S_herbacea_marPrecip	marPrecip	1.39	1.41	0.1554551	
S_herbacea_marPrecip	NHMD_ID	14.76	26.15	6.33334E-05	
S_herbacea_marPrecip	NHMD_ID/STEM	24.28	2.63	0.259679732	
S_herbacea_aprPrecip	aprPrecip	1.62	0.45	0.592302736	
S_herbacea_aprPrecip	NHMD_ID	16.24	22.64	0	
S_herbacea_aprPrecip	NHMD_ID/STEM	20.21	1.49	0.213395459	
S_herbacea_mayPrecip	mayPrecip	1.00	0.42	0.516462064	
S_herbacea_mayPrecip	NHMD_ID	16.22	22.71	0	
S_herbacea_mayPrecip	NHMD_ID/STEM	20.03	1.51	0.236977106	
S_herbacea_junPrecip	junPrecip	1.95	1.53	0.198029503	
S_herbacea_junPrecip	NHMD_ID	16.83	24.24	0	
S_herbacea_junPrecip	NHMD_ID/STEM	19.56	1.32	0.359273547	
S_herbacea_julPrecip	julPrecip	1.68	2.99	0.052433021	
S_herbacea_julPrecip	NHMD_ID	16.21	28.30	0	
S_herbacea_julPrecip	NHMD_ID/STEM	22.02	1.86	0.271470292	

S_herbacea_augPrecip	augPrecip	4.62	1.01	0.460611052	
S_herbacea_augPrecip	NHMD_ID	16.03	21.67	0	
S_herbacea_augPrecip	NHMD_ID/STEM	19.70	1.38	0.171099988	
S_herbacea_annualPrecip	annualPrecip	2.50	0.62	0.616468825	
S_herbacea_annualPrecip	NHMD_ID	15.80	23.31	4.43387E-07	
S_herbacea_annualPrecip	NHMD_ID/STEM	19.80	1.38	0.336733528	
**S_herbacea_growth_year**	**growth_year**	**3.65**	**3.72**	**0.010460992**	*
S_herbacea_growth_year	NHMD_ID	11.67	13.56	0.010468986	
S_herbacea_growth_year	NHMD_ID/STEM	29.82	2.62	0.187961971	
**S_glauca_julAveTemp**	**julAveTemp**	**2.65**	**3.40**	**0.02081539**	*
S_glauca_julAveTemp	NHMD_ID	7.22	22.49	0.000528405	
S_glauca_julAveTemp	NHMD_ID/STEM	17.15	4.98	0.028495874	
S_glauca_junAveTemp	junAveTemp	1.00	0.15	0.695870619	
S_glauca_junAveTemp	NHMD_ID	6.20	12.29	0.001521461	
S_glauca_junAveTemp	NHMD_ID/STEM	16.94	4.21	0.008245427	
S_glauca_mayAveTemp	mayAveTemp	8.29	2.01	0.060925297	
S_glauca_mayAveTemp	NHMD_ID	8.85	17.69	0.000230859	
S_glauca_mayAveTemp	NHMD_ID/STEM	16.87	3.25	0.024732373	
**S_glauca_aprAveTemp**	**aprAveTemp**	**8.05**	**2.55**	**0.019828678**	*
S_glauca_aprAveTemp	NHMD_ID	7.70	19.33	0.000516515	
S_glauca_aprAveTemp	NHMD_ID/STEM	20.07	5.07	0.005827135	
S_glauca_marAveTemp	marAveTemp	3.26	1.37	0.252759773	
S_glauca_marAveTemp	NHMD_ID	7.70	13.85	0.000637745	
S_glauca_marAveTemp	NHMD_ID/STEM	18.79	3.82	0.008492218	
S_glauca_febAveTemp	febAveTemp	1.00	1.52	0.22200106	
S_glauca_febAveTemp	NHMD_ID	6.81	11.49	0.000900183	
S_glauca_febAveTemp	NHMD_ID/STEM	18.89	3.55	0.007859677	
S_glauca_janAveTemp	janAveTemp	1.00	2.60	0.111092539	
S_glauca_janAveTemp	NHMD_ID	8.19	14.32	0.002166954	
S_glauca_janAveTemp	NHMD_ID/STEM	18.25	3.57	0.043478761	
**S_glauca_augAveTemp**	**augAveTemp**	**1.00**	**6.38**	**0.014012926**	*
S_glauca_augAveTemp	NHMD_ID	7.52	18.22	0.001198289	
S_glauca_augAveTemp	NHMD_ID/STEM	19.16	4.62	0.026657965	
S_glauca_octAveTemp.1	octAveTemp.1	1.00	0.29	0.591312114	
S_glauca_octAveTemp.1	NHMD_ID	7.18	11.36	0.001199797	
S_glauca_octAveTemp.1	NHMD_ID/STEM	18.53	3.49	0.010154186	
S_glauca_novAveTemp.1	novAveTemp.1	1.00	0.44	0.508818427	
S_glauca_novAveTemp.1	NHMD_ID	7.18	11.24	0.000867217	
S_glauca_novAveTemp.1	NHMD_ID/STEM	18.51	3.50	0.007482297	
S_glauca_decAveTemp.1	decAveTemp.1	6.03	1.51	0.13270096	
S_glauca_decAveTemp.1	NHMD_ID	7.49	9.75	0.00471784	
S_glauca_decAveTemp.1	NHMD_ID/STEM	18.94	3.98	0.006189961	
S_glauca_annualAveTemp	annualAveTemp	1.94	1.32	0.419288912	
S_glauca_annualAveTemp	NHMD_ID	6.15	10.76	0.001113543	
S_glauca_annualAveTemp	NHMD_ID/STEM	16.86	3.88	0.006885731	

**S_glauca_Anomaly**	**Anomaly**	**7.58**	**2.94**	**0.008359556**	**
S_glauca_Anomaly	NHMD_ID	5.68	17.90	7.36886E-05	
S_glauca_Anomaly	NHMD_ID/STEM	17.75	5.92	0.000386375	
**S_glauca_annualAveTemp.1**	**annualAveTemp.1**	**1.00**	**10.35**	**0.002031141**	**
S_glauca_annualAveTemp.1	NHMD_ID	4.91	7.68	0.005032766	
S_glauca_annualAveTemp.1	NHMD_ID/STEM	17.87	4.25	0.002088863	
**S_glauca_Anomaly.1**	**Anomaly.1**	**1.00**	**5.16**	**0.02641261**	*
S_glauca_Anomaly.1	NHMD_ID	6.80	13.97	0.001578567	
S_glauca_Anomaly.1	NHMD_ID/STEM	16.35	4.63	0.009534383	
S_glauca_janPrecip	janPrecip	6.21	3.93	0.001603074	
S_glauca_janPrecip	NHMD_ID	8.56	47.70	6.57152E-05	
S_glauca_janPrecip	NHMD_ID/STEM	16.87	6.25	0.04039664	
S_glauca_febPrecip	febPrecip	1.00	6.44	0.01376491	
S_glauca_febPrecip	NHMD_ID	5.73	12.43	0.00171695	
S_glauca_febPrecip	NHMD_ID/STEM	17.71	4.54	0.006022665	
S_glauca_marPrecip	marPrecip	5.88	2.93	0.010851835	
S_glauca_marPrecip	NHMD_ID	7.74	25.78	0.000227065	
S_glauca_marPrecip	NHMD_ID/STEM	16.59	4.74	0.02604478	
S_glauca_aprPrecip	aprPrecip	1.00	1.09	0.301341054	
S_glauca_aprPrecip	NHMD_ID	5.87	15.46	0.000691486	
S_glauca_aprPrecip	NHMD_ID/STEM	16.45	4.56	0.009515634	
S_glauca_mayPrecip	mayPrecip	4.56	4.20	0.002423282	
S_glauca_mayPrecip	NHMD_ID	5.82	16.51	0.000929963	
S_glauca_mayPrecip	NHMD_ID/STEM	17.26	5.29	0.005448527	
S_glauca_junPrecip	junPrecip	1.00	3.00	0.088999504	
S_glauca_junPrecip	NHMD_ID	6.68	23.95	0.00100172	
S_glauca_junPrecip	NHMD_ID/STEM	16.82	5.16	0.044595177	
S_glauca_julPrecip	julPrecip	1.00	1.37	0.247341345	
S_glauca_julPrecip	NHMD_ID	5.99	15.11	0.00183444	
S_glauca_julPrecip	NHMD_ID/STEM	16.81	4.89	0.012553003	
S_glauca_augPrecip	augPrecip	1.00	1.99	0.163775937	
S_glauca_augPrecip	NHMD_ID	6.06	15.84	0.001524928	
S_glauca_augPrecip	NHMD_ID/STEM	16.91	5.33	0.009692269	
S_glauca_annualPrecip	annualPrecip	1.46	2.71	0.135731386	
S_glauca_annualPrecip	NHMD_ID	5.95	19.60	0.000310764	
S_glauca_annualPrecip	NHMD_ID/STEM	16.99	5.35	0.004139133	
S_glauca_growth_year	growth_year	7.86	1.55	0.153406571	
S_glauca_growth_year	NHMD_ID	10.71	29.22	0.002019219	
S_glauca_growth_year	NHMD_ID/STEM	14.78	3.88	0.136233748	

Table S3. Variance explained by predictor variables and random intercept effects of (NHMD_ID) and (NHMD_ID/STEM) for each fitted GAM model (models fitted separately for each species and each predictor variable).

Model	Variance		
	s.julAveTemp.	s.NHMD_ID.	s.NHMD_ID.branch_No.
S_arctica_julAveTemp	0.001	0.564	0.251
S_arctica_junAveTemp	0.001	0.558	0.250
S_arctica_mayAveTemp	0.000	0.555	0.254
S_arctica_aprAveTemp	0.000	0.557	0.255
S_arctica_marAveTemp	0.000	0.553	0.254
S_arctica_febAveTemp	0.029	0.519	0.255
S_arctica_janAveTemp	0.000	0.556	0.254
S_arctica_augAveTemp	0.001	0.559	0.255
S_arctica_octAveTemp.1	0.028	0.559	0.251
S_arctica_novAveTemp.1	0.000	0.554	0.255
S_arctica_decAveTemp.1	0.000	0.552	0.254
S_arctica_annualAveTemp	0.000	0.564	0.255
S_arctica_Anomaly	0.020	0.549	0.256
S_arctica_annualAveTemp.1	0.001	0.554	0.254
S_arctica_Anomaly.1	0.001	0.546	0.255
S_arctica_janPrecip	0.002	0.542	0.238
S_arctica_febPrecip	0.000	0.645	0.264
S_arctica_marPrecip	0.000	0.654	0.263
S_arctica_aprPrecip	0.001	0.545	0.240
S_arctica_mayPrecip	0.000	0.571	0.238
S_arctica_junPrecip	0.000	0.569	0.238
S_arctica_julPrecip	0.000	0.583	0.229
S_arctica_augPrecip	0.000	0.567	0.237
S_arctica_annualPrecip	0.000	0.705	0.280
S_arctica_growth_year	0.020	1.042	0.264
S_herbacea_julAveTemp	0.603	0.401	0.295
S_herbacea_junAveTemp	0.514	0.391	0.293
S_herbacea_mayAveTemp	0.079	0.430	0.300
S_herbacea_aprAveTemp	0.000	0.434	0.299
S_herbacea_marAveTemp	0.000	0.424	0.300
S_herbacea_febAveTemp	0.000	0.433	0.298
S_herbacea_janAveTemp	0.007	0.389	0.300
S_herbacea_augAveTemp	2.356	0.378	0.313
S_herbacea_octAveTemp.1	0.312	0.439	0.308
S_herbacea_novAveTemp.1	0.029	0.422	0.295
S_herbacea_decAveTemp.1	0.000	0.423	0.300
S_herbacea_annualAveTemp	0.228	0.448	0.307
S_herbacea_Anomaly	0.000	0.640	0.307
S_herbacea_annualAveTemp.1	0.556	0.431	0.306

S_herbacea_Anomaly.1	0.100	0.408	0.302
S_herbacea_janPrecip	0.007	0.422	0.283
S_herbacea_febPrecip	0.003	0.432	0.301
S_herbacea_marPrecip	0.002	0.423	0.301
S_herbacea_aprPrecip	0.001	0.476	0.249
S_herbacea_mayPrecip	0.000	0.471	0.246
S_herbacea_junPrecip	0.001	0.516	0.241
S_herbacea_julPrecip	0.001	0.489	0.270
S_herbacea_augPrecip	0.010	0.451	0.238
S_herbacea_annualPrecip	0.001	0.482	0.256
S_herbacea_growth_year	0.009	0.330	0.324
S_glauca_julAveTemp	0.258	0.399	0.447
S_glauca_junAveTemp	0.001	0.334	0.424
S_glauca_mayAveTemp	3.312	0.439	0.402
S_glauca_aprAveTemp	1.304	0.381	0.439
S_glauca_marAveTemp	0.040	0.365	0.408
S_glauca_febAveTemp	0.000	0.317	0.396
S_glauca_janAveTemp	0.000	0.385	0.403
S_glauca_augAveTemp	0.001	0.381	0.447
S_glauca_octAveTemp.1	0.000	0.333	0.393
S_glauca_novAveTemp.1	0.000	0.331	0.393
S_glauca_decAveTemp.1	0.224	0.365	0.414
S_glauca_annualAveTemp	0.119	0.333	0.421
S_glauca_Anomaly	1.717	0.342	0.448
S_glauca_annualAveTemp.1	0.000	0.271	0.417
S_glauca_Anomaly.1	0.000	0.364	0.413
S_glauca_janPrecip	0.148	0.495	0.461
S_glauca_febPrecip	0.000	0.320	0.439
S_glauca_marPrecip	0.139	0.427	0.433
S_glauca_aprPrecip	0.000	0.339	0.435
S_glauca_mayPrecip	0.028	0.340	0.445
S_glauca_junPrecip	0.000	0.394	0.464
S_glauca_julPrecip	0.000	0.347	0.446
S_glauca_augPrecip	0.000	0.351	0.449
S_glauca_annualPrecip	0.000	0.345	0.446
S_glauca_growth_year	0.101	1.214	0.445
S_arctophila_julAveTemp	0.037	0.382	0.353
S_arctophila_junAveTemp	0.000	0.378	0.359
S_arctophila_mayAveTemp	0.079	0.394	0.361
S_arctophila_aprAveTemp	0.018	0.376	0.362
S_arctophila_marAveTemp	0.020	0.371	0.364
S_arctophila_febAveTemp	0.011	0.367	0.357
S_arctophila_janAveTemp	0.000	0.405	0.355
S_arctophila_augAveTemp	0.145	0.373	0.359
S_arctophila_octAveTemp.1	0.000	0.365	0.361

S_arctophila_novAveTemp.1	0.000	0.362	0.356
S_arctophila_decAveTemp.1	0.032	0.366	0.360
S_arctophila_annualAveTemp	0.001	0.421	0.359
S_arctophila_Anomaly	0.095	0.372	0.361
S_arctophila_annualAveTemp.1	0.054	0.331	0.366
S_arctophila_Anomaly.1	1.796	0.248	0.382
S_arctophila_janPrecip	0.000	0.297	0.387
S_arctophila_febPrecip	0.001	0.276	0.374
S_arctophila_marPrecip	0.000	0.267	0.374
S_arctophila_aprPrecip	0.002	0.273	0.399
S_arctophila_mayPrecip	0.038	0.242	0.408
S_arctophila_junPrecip	0.000	0.292	0.393
S_arctophila_julPrecip	0.000	0.287	0.403
S_arctophila_augPrecip	0.000	0.275	0.409
S_arctophila_annualPrecip	0.000	0.316	0.386
S_arctophila_growth_year	0.000	0.374	0.358

Table S4. Model metrics from Generalized Additive Models (GAMs) run for each Salix species with climatic or temporal variables as predictors and individual (specimen ID) and specimen ID/stem as random intercepts.

Species	Predictor	Adjusted_R2	Deviance_Explained	GCV	Sample_Size
S_arctica	julAveTemp	0.78	77.93	0.25	189
S_arctica	junAveTemp	0.78	77.92	0.25	189
S_arctica	mayAveTemp	0.78	77.89	0.25	189
S_arctica	aprAveTemp	0.78	77.91	0.25	189
S_arctica	marAveTemp	0.78	77.87	0.25	189
S_arctica	febAveTemp	0.79	78.58	0.25	189
S_arctica	janAveTemp	0.78	77.87	0.25	189
S_arctica	augAveTemp	0.78	77.90	0.25	189
S_arctica	octAveTemp.1	0.79	78.10	0.25	189
S_arctica	novAveTemp.1	0.78	77.90	0.25	189
S_arctica	decAveTemp.1	0.78	78.32	0.25	189
S_arctica	annualAveTemp	0.78	77.89	0.25	189
S_arctica	Anomaly	0.78	78.00	0.25	189
S_arctica	annualAveTemp.1	0.78	77.87	0.25	189
S_arctica	Anomaly.1	0.78	77.94	0.25	189
S_arctica	janPrecip	0.78	77.81	0.25	185
S_arctica	febPrecip	0.78	78.80	0.24	176
S_arctica	marPrecip	0.79	78.76	0.24	176
S_arctica	aprPrecip	0.79	78.06	0.25	179
S_arctica	mayPrecip	0.78	77.56	0.26	179
S_arctica	junPrecip	0.79	77.50	0.26	179
S_arctica	julPrecip	0.79	77.76	0.26	176
S_arctica	augPrecip	0.79	77.50	0.26	179
S_arctica	annualPrecip	0.80	79.45	0.23	167

S_arctica	growth_year	0.80	79.56	0.24	189
S_herbacea	julAveTemp	0.45	64.55	0.19	266
S_herbacea	junAveTemp	0.49	65.90	0.18	266
S_herbacea	mayAveTemp	0.45	64.06	0.19	266
S_herbacea	aprAveTemp	0.46	64.43	0.18	266
S_herbacea	marAveTemp	0.44	63.89	0.19	266
S_herbacea	febAveTemp	0.44	64.18	0.19	266
S_herbacea	janAveTemp	0.50	66.26	0.18	266
S_herbacea	augAveTemp	0.49	66.52	0.18	266
S_herbacea	octAveTemp.1	0.46	65.53	0.19	266
S_herbacea	novAveTemp.1	0.50	64.94	0.18	266
S_herbacea	decAveTemp.1	0.45	64.25	0.19	266
S_herbacea	annualAveTemp	0.45	65.69	0.18	266
S_herbacea	Anomaly	0.47	65.49	0.18	266
S_herbacea	annualAveTemp.1	0.48	66.54	0.18	266
S_herbacea	Anomaly.1	0.44	64.02	0.19	266
S_herbacea	janPrecip	0.48	65.97	0.19	242
S_herbacea	febPrecip	0.50	66.49	0.19	227
S_herbacea	marPrecip	0.51	66.62	0.19	227
S_herbacea	aprPrecip	0.48	66.56	0.19	217
S_herbacea	mayPrecip	0.48	66.37	0.19	216
S_herbacea	junPrecip	0.48	67.15	0.19	217
S_herbacea	julPrecip	0.51	71.07	0.18	199
S_herbacea	augPrecip	0.49	67.18	0.19	217
S_herbacea	annualPrecip	0.55	72.15	0.19	182
S_herbacea	growth_year	0.47	65.71	0.18	266
S_glauca	julAveTemp	0.65	77.07	0.26	89
S_glauca	junAveTemp	0.63	71.36	0.29	89
S_glauca	mayAveTemp	0.68	78.15	0.29	93
S_glauca	aprAveTemp	0.65	77.52	0.26	103
S_glauca	marAveTemp	0.61	71.66	0.28	103
S_glauca	febAveTemp	0.58	68.73	0.28	103
S_glauca	janAveTemp	0.62	70.23	0.28	103
S_glauca	augAveTemp	0.61	75.39	0.26	93
S_glauca	octAveTemp.1	0.61	68.38	0.29	103
S_glauca	novAveTemp.1	0.62	68.37	0.29	103
S_glauca	decAveTemp.1	0.64	74.00	0.28	103
S_glauca	annualAveTemp	0.65	72.62	0.29	89
S_glauca	Anomaly	0.70	80.10	0.26	89
S_glauca	annualAveTemp.1	0.66	73.44	0.27	89
S_glauca	Anomaly.1	0.67	73.52	0.27	89
S_glauca	janPrecip	0.74	82.48	0.24	85
S_glauca	febPrecip	0.62	74.32	0.28	85
S_glauca	marPrecip	0.70	80.55	0.26	85
S_glauca	aprPrecip	0.70	75.44	0.28	79
S_glauca	mayPrecip	0.73	82.57	0.23	79
S_glauca	junPrecip	0.72	77.66	0.27	79
S_glauca	julPrecip	0.71	76.11	0.28	79
S_glauca	augPrecip	0.71	76.56	0.27	79
S_glauca	annualPrecip	0.72	77.46	0.27	79
S_glauca	growth_year	0.60	75.55	0.27	103

S_arctophila	julAveTemp	0.47	66.48	0.31	222
S_arctophila	junAveTemp	0.47	65.97	0.32	222
S_arctophila	mayAveTemp	0.48	67.06	0.32	224
S_arctophila	aprAveTemp	0.49	68.10	0.30	227
S_arctophila	marAveTemp	0.48	66.96	0.31	227
S_arctophila	febAveTemp	0.50	66.47	0.31	227
S_arctophila	janAveTemp	0.47	65.88	0.32	227
S_arctophila	augAveTemp	0.49	67.29	0.31	222
S_arctophila	octAveTemp.1	0.47	65.76	0.32	227
S_arctophila	novAveTemp.1	0.47	65.45	0.32	227
S_arctophila	decAveTemp.1	0.48	66.73	0.32	227
S_arctophila	annualAveTemp	0.48	66.36	0.32	222
S_arctophila	Anomaly	0.50	67.64	0.31	222
S_arctophila	annualAveTemp.1	0.50	66.40	0.31	222
S_arctophila	Anomaly.1	0.54	70.26	0.30	222
S_arctophila	janPrecip	0.49	65.70	0.30	206
S_arctophila	febPrecip	0.53	66.27	0.29	197
S_arctophila	marPrecip	0.53	65.97	0.29	197
S_arctophila	aprPrecip	0.51	66.67	0.30	198
S_arctophila	mayPrecip	0.54	68.03	0.31	198
S_arctophila	junPrecip	0.49	65.54	0.31	198
S_arctophila	julPrecip	0.52	67.07	0.30	197
S_arctophila	augPrecip	0.51	66.80	0.30	198
S_arctophila	annualPrecip	0.55	67.13	0.30	188
S_arctophila	growth_year	0.47	65.48	0.32	227

Table S5. Spearman Rank Correlation Analyses of the annual growth increments measured on the first and second stems from each digital specimens for the four *Salix* species

Species	Correlation Co-efficient	p-value	Number of stem pairs (n)
Salix arctica	0.92	**> 0.001**	16
Salix arctophila	0.43	0.06	22
Salix glauca	0.31	0.56	7
Salix herbacea	0.68	**> 0.01**	18

Table S6. Spearman Rank Correlation Analyses of annual growth increment and mean temperature in July corresponding to the year of growth

Species	Correlation Co-efficient	p-value	Number of years (n)
Salix arctica	-0.38	**0.03**	33
Salix arctophila	-0.03	0.82	59
Salix glauca	0.18	0.32	31
Salix herbacea	-0.02	0.91	44

Figures

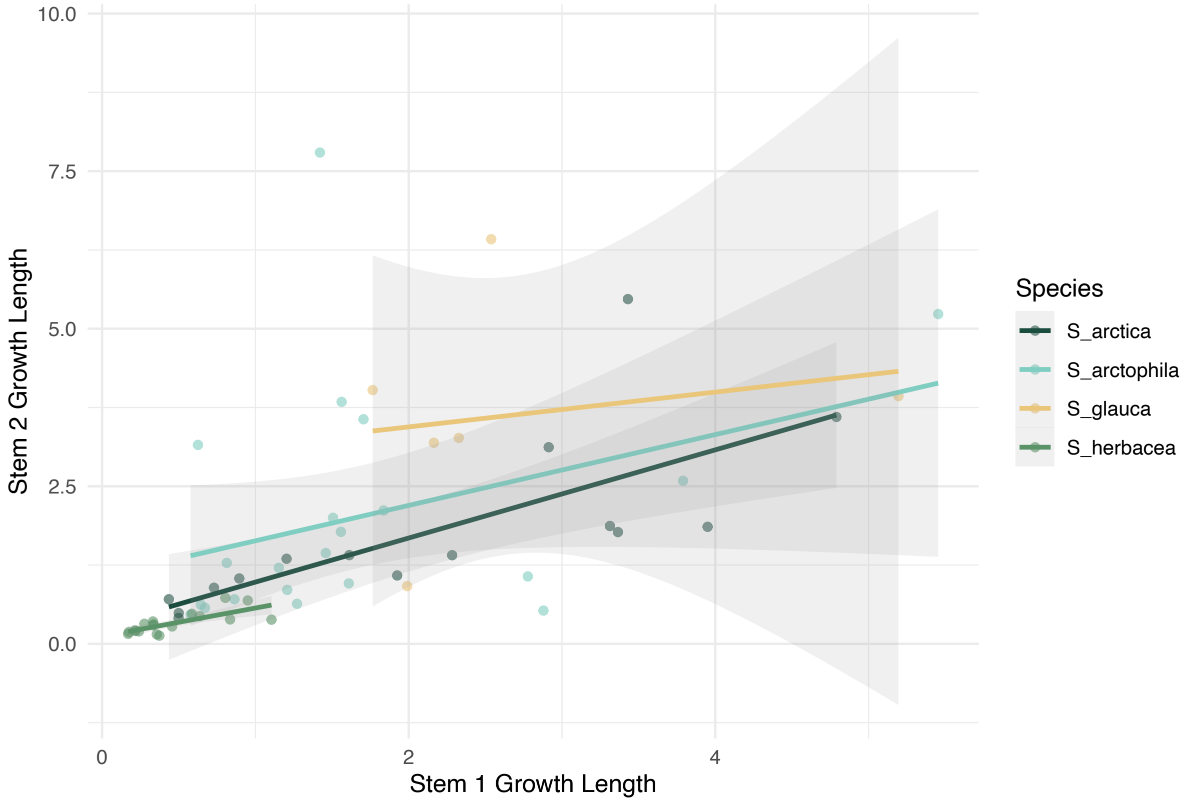

Figure S1. Relationship between annual growth increments on the first and second stems from the same specimens (NHMD_ID) for *Salix* species

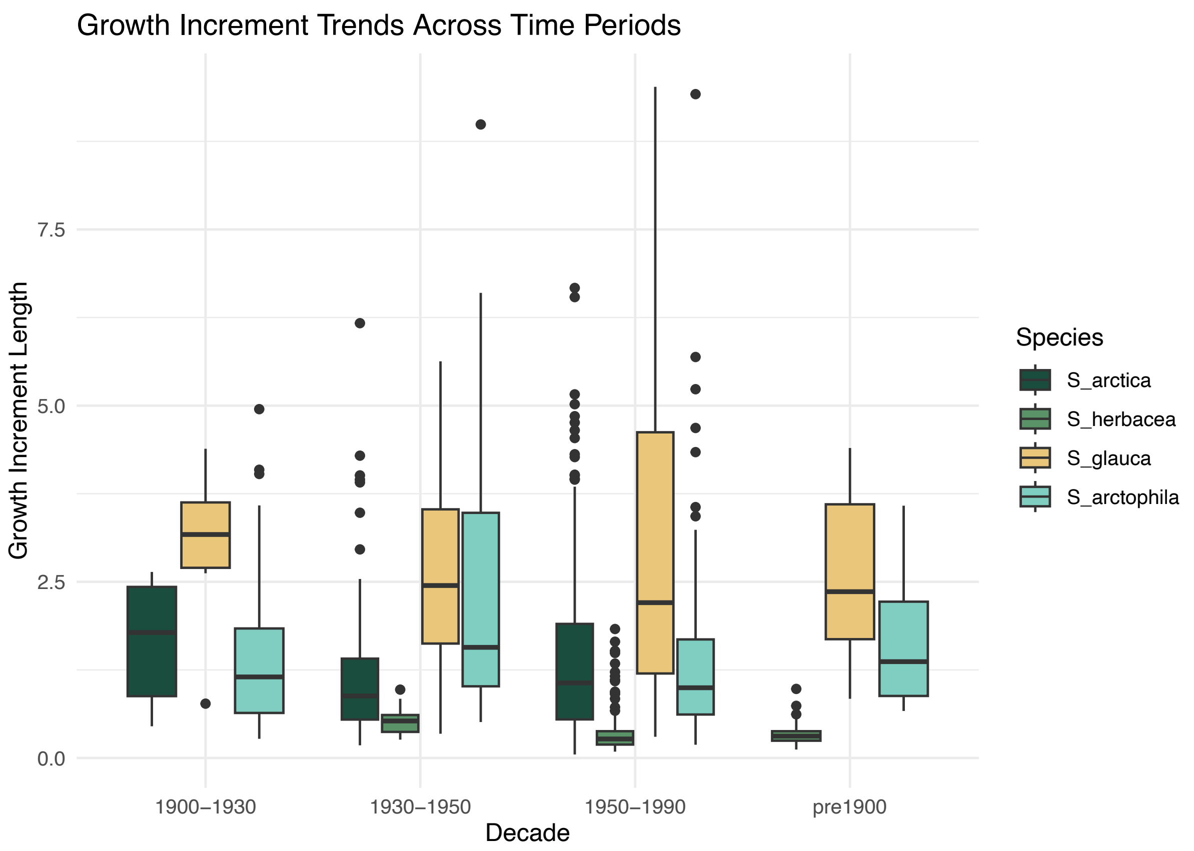

Figure S2. Variability in growth increments over time for *Salix* species collected in the Disko Bay phytogeographic region of Western Greenland.
